# Genome-wide fine-mapping improves identification of causal variants

**DOI:** 10.21203/rs.3.rs-4759390/v1

**Published:** 2024-08-07

**Authors:** Yang Wu, Zhili Zheng, Loic Thibaut2, Michael E. Goddard, Naomi R. Wray, Peter M. Visscher, Jian Zeng

**Affiliations:** 1 Institute of Rare Diseases, West China Hospital of Sichuan University, Chengdu, China; 2 Institute for Molecular Bioscience, The University of Queensland, Brisbane, Queensland, Australia; 3 Program in Medical and Population Genetics, Broad Institute of Harvard and MIT, Cambridge, Massachusetts, USA; 4 Stanley Center for Psychiatric Research, Broad Institute of Harvard and MIT, Cambridge, Massachusetts, USA; 5 Faculty of Veterinary and Agricultural Science, University of Melbourne, Parkville, Victoria, Australia; 6 Biosciences Research Division, Department of Economic Development, Jobs, Transport and Resources, Bundoora, Victoria, Australia; 7 Department of Psychiatry, University of Oxford, Oxford, UK; 8 Big Data Institute, Li Ka Shing Centre for Health Information and Discovery, Nuffield Department of Population Health, University of Oxford, Oxford, UK

## Abstract

Fine-mapping refines genotype-phenotype association signals to identify causal variants underlying complex traits. However, current methods typically focus on individual genomic segments without considering the global genetic architecture. Here, we demonstrate the advantages of performing genome-wide fine-mapping (GWFM) and develop methods to facilitate GWFM. In simulations and real data analyses, GWFM outperforms current methods in error control, mapping power and precision, replication rate, and trans-ancestry phenotype prediction. For 48 well-powered traits in the UK Biobank, we identify causal variants that collectively explain 17% of the SNP-based heritability, and predict that fine-mapping 50% of that would require 2 million samples on average. We pinpoint a known causal variant, as proof-of-principle, at FTO for body mass index, unveil a hidden secondary variant with evolutionary conservation, and identify new missense causal variants for schizophrenia and Crohn’s disease. Overall, we analyse 600 complex traits with 13 million SNPs, highlighting the efficacy of GWFM with functional annotations.

## Introduction

Genome-wide association studies (GWAS) have successfully identified numerous genetic variants associated with complex traits^1–3^. However, the underlying casual variants for these traits are largely unknown. In a standard GWAS, the trait-variant associations are tested one at a time, leading to the discovery of clusters of mutually correlated marginal-association signals due to linkage disequilibrium (LD) between SNPs^4^. While post-GWAS methods such as LD clumping^5^ or COJO^6^ are used to identify independently significant association signals, SNPs prioritised by these methods are not necessarily the causal variants^7,8^.

Statistical fine-mapping, often employing a Bayesian mixture model (BMM), offers a direct approach to narrow down the likely causal variants^9^. In contrast to GWAS, which test marginal effects, fine-mapping aims to detect joint-association signals for causal inference, where the strength of joint association is assessed using the posterior inclusion probability (PIP). PIP is the probability of a SNP being included with a nonzero effect in the model, which, in theory, controls false discovery rate (FDR)^10^. Due to the computational burden and complexity of test hypotheses, current fine-mapping methods focus on genome-wide significant loci only or consider one genomic region at a time (e.g., a LD block), in isolation from the rest of the genome^11–14^. Methods differ mainly in the algorithm used to derive PIP. For example, FINEMAP^12^ utilizes a shotgun stochastic search algorithm to explore possible causal configurations, and computes the PIP by averaging over those with non-negligible probabilities. SuSiE^11^ and SuSiE-RSS^14^ assume a sparse effect model and employ an iterative Bayesian stepwise selection approach to estimate the overall effect of each SNP by summing up multiple singe-effect vectors. SuSiE-Inf^13^ and FINEMAP-Inf^13^ further extend the two models to include an infinitesimal component for improved modelling of polygenic architecture within a locus.

Despite being widely used, region-specific analysis has limitations. First, the prior specification of genetic architecture is crucial, but is often conservatively predetermined in these analysis^11,12,15^ (e.g., prior probability of association set to one over the number of SNPs in the region), which can result in reduced power. Second, fine-mapping can benefit from incorporating functional genomic annotations^16–18^, but region-specific methods require step-wise procedures so that GWAS data and functional annotations are not modelled jointly^19^. Third, none of the current methods estimates the power of identifying the causal variants for a trait, which is critical to inform the experimental design of a prospective study (such a power analysis is available in GWAS^20^ but not in fine-mapping).

These limitations of current fine-mapping methods can be addressed through conducting a fine-mapping analysis using a genome-wide Bayesian mixture model (GBMM). GBMMs, which have been widely used for predicting breeding values in agricultural species^21–23^ and complex trait phenotypes in humans^24–27^, have recently emerged as a method of GWFM^28,29^. Compared to conventional GWAS and region-specific fine-mapping approaches, GBMMs consider genome-wide SNPs simultaneously, which are all utilised to estimate the genetic architecture and functional prior^27,28^. For example, SNPs with the same class of functional annotation are present across the genome. By considering all SNPs jointly, the importance of a functional annotation in a local genomic region can be prioritised based on the evidence for association as a class across the genome. In GBMMs, Markov chain Monte Carlo (MCMC) sampling is often used for posterior inference, which is asymptotically exact and superior to the variational inference regarding accuracy^30^, but computationally challenging when analysing high-density SNPs. Fortunately, recent advances in methodology, such as SBayesRC^27^, have allowed fitting all common SNPs efficiently in a MCMC-based GBMM. Moreover, GBMMs estimate the polygenicity and variant effect size distribution^22,24,26,27,31,32^, providing an opportunity to predict the power of prospective studies with larger sample sizes. However, relevant theory and methods have not yet been developed.

**Figure 1:**
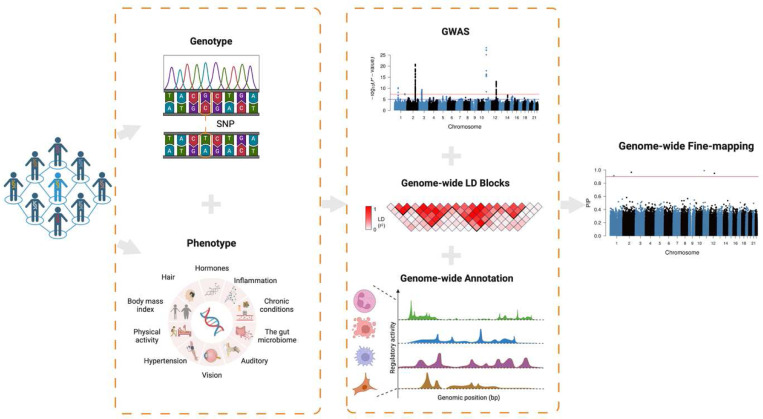
Schematic overview of genome-wide fine-mapping analysis using GBMM. GBMM requires the GWAS summary statistics and genome-wide LD reference to fine-map the likely causal variants for complex traits, and can incorporate functional annotations. Compared to regional-based fine-mapping approaches, GBMM estimates priors with genome-wide SNPs and MCMC sampling algorithm, and is more flexible on the assumption of the underlying distribution of causal effects (**Table S1**). The illustration was created with BioRender.com.

In this study, we comprehensively assess the performance of GWFM analysis using a GBMM (Fig. 1). In comparison to state-of-the-art methods, we evaluate the calibration of PIP through simulations with various genetic architecture settings. We then compare the performance of identifying causal variants, with respect to mapping precision, credible set size, replication rate of discovery in an independent sample, and out-of-sample prediction using fine-mapped variants. Moreover, we develop a LD-based method to construct local credible sets (LCSs), where a α-LCS represents a minimal set of SNPs in high LD that capture a causal variant with a posterior probability of α, and estimate the proportion of SNP-based heritability explained by LCSs. To characterise the overall fine-mapping power in the current study, we propose a concept of global credible sets (GCSs), with a α-GCS representing a minimal set of genome-wide SNPs that capture α% of all causal variants for the trait. Furthermore, leveraging the genetic architecture estimated from SBayesRC, we develop a method to predict the power of fine-mapping and variance explained by the identified variants in prospective studies. With this method, we can estimate the minimal sample size required for identifying a desired proportion of causal variants or those variants explaining a desired proportion of the SNP-based heritability ($h_{SNP}^{2}$). Finally, we apply SBayesRC to the UK Biobank (UKB) data with 13 million SNPs to identify putative causal variants for 600 complex traits and diseases and compare the fine-mapping results using 48 well-powered traits from 6 categories.

## Results

### Method overview

We selected SBayesRC as the GBMM for GWFM (Fig. 1), as it has been shown to outperform other GBMMs in polygenic prediction^27^. SBayesRC is a hierarchical multi-component mixture model, where LD between SNPs is better modelled by matrix factorisation and functional genomic annotations are fitted jointly with the summary statistics in a unified computational framework (Methods). To optimize its performance for fine-mapping, we implemented an algorithm to automatically determine the number of mixture components in the model (Methods). In contrast to the existing fine-mapping methods, we fit all SNPs simultaneously and employed MCMC sampling to obtain the joint posterior distribution of model parameters and PIPs (**Supplementary Table 1**). In each MCMC iteration, we sampled a dummy variable for each SNP to indicate whether the SNP had a nonzero effect, conditional on the effects of other SNPs. After MCMC sampling, PIP was calculated as the frequency with which the SNP had nonzero effects across the iterations (Methods).

A high PIP value provides evidence of a causal variant. However, a causal variant may not have a high PIP value if it is in strong LD with other SNPs. For example, if the causal variant is in perfect LD with another SNP, then the PIP is expected to be 0.5 for each variant, regardless of the sample size. Therefore, the CS concept has been introduced to capture causal variants in strong LD with non-causal SNPs^9,33^. It is common to consider CS for SNPs that are close in physical distance, such as within a 100kb window^34,35^. However, we reason that this approach will miss causal variants with SNPs in long-range LD, and therefore proposed a new method to construct LCS based on LD between SNPs (Methods). Starting from the SNP with the largest PIP, we aimed to construct a α-LCS for each “free” SNP (SNP that has not been included in any LCSs), by first selecting other “free” SNPs in high LD (*r^2^* > 0.5) and then summing over their PIPs in a decreasing order until the sum is at least α (a common strategy used in the literature^11,12^). To avoid having too many SNPs with small PIPs in the LCS, we calculated the posterior $h_{SNP}^{2}$ enrichment probability (PEP), where PEP is the probability that the focal LCS explains more $h_{SNP}^{2}$ than a random set of SNPs with the same size. The α-LCS was eventually reported if its PEP was greater than 0.7.

In addition to LCS, we proposed another type of CS, GCS. Given the estimated number of causal variants from GBMM ($m_{c}$), a α-GCS was computed as the cumulative sum of decreasingly ranked PIPs that is greater than $\alpha \times m_{c}$. It can be shown that a α-GCS is expected to cover α% of all causal variants for the trait (Methods), with the size of α-GCS reflecting the power of identifying the causal variants given the data (the higher the power, the smaller the α-GCS size). Moreover, from the MCMC samples of SNP effects, we estimated the proportion of $h_{SNP}^{2}$ explained by the LCSs and GCS (Methods).

Based on $m_{c}$ and the distribution of causal effect sizes estimated from GBMM, we developed a method to predict the power and the proportion of $h_{SNP}^{2}$ explained by the fine-mapped variants, given a sample size (Methods and **Supplementary Note**). This method allows us to estimate the minimal sample size required to achieve a desired power of identifying all causal variants or identifying the causal variants that explain a desired proportion of $h_{SNP}^{2}$ of the trait, using the ancestry-specific fine-mapping result. Our method to predict fine-mapping power is analytically tractable and has been implemented in a publicly available online tool (https://sbayes.pctgplots.cloud.edu.au/shiny/power/).

We compared SBayesRC to several state-of-the-art fine-mapping methods, including FINEMAP^12^, SuSiE^11^, FINEMAP-inf^13^, SuSiE-inf^7^, and PolyFun+SuSiE^19^, as well as another GBMM, SBayesC (i.e., two-component SBayesR^24^). All these methods assume a point-normal mixture prior for the SNP effects (Methods and **Supplementary Table 1**). A full list of acronyms used in this study can be found in **Supplementary Table 2**.

### Calibration of fine-mapping methods under various genetic architectures

We performed extensive genome-wide simulations to calibrate different fine-mapping methods under various genetic architectures, using 100,000 individuals with ~1 million HapMap3 SNPs from the UKB^36^. We started by simulating a sparse genetic architecture, where 1% SNPs were randomly chosen as causal variants, with their effects sampled from a normal distribution, contributing 50% of the phenotypic variance. In this simulation, the data-generative model is consistent with the model used in SBayesC as well as the local fine-mapping methods in comparison. To challenge these methods, we simulated additional two complex genetic architectures (Methods). One was a large-effects architecture, where 10 random causal variants contributed 10% of the phenotypic variance and the remaining causal variants contributed 40%. Another complex genetic architecture was based on the sparse architecture but allowed for extensive LD between causal variants and SNP markers. This was achieved by sampling the causal variants only from SNPs in the high LD and high minor allele frequency (MAF) group, and therefore referred to as LD-and-MAF-stratified (LDMS) architecture. To calibrate each fine-mapping method, we evaluated how well the reported PIPs are consistent with the actual fraction of causal variants, i.e., the true discovery rate (TDR).

**Figure 2:**
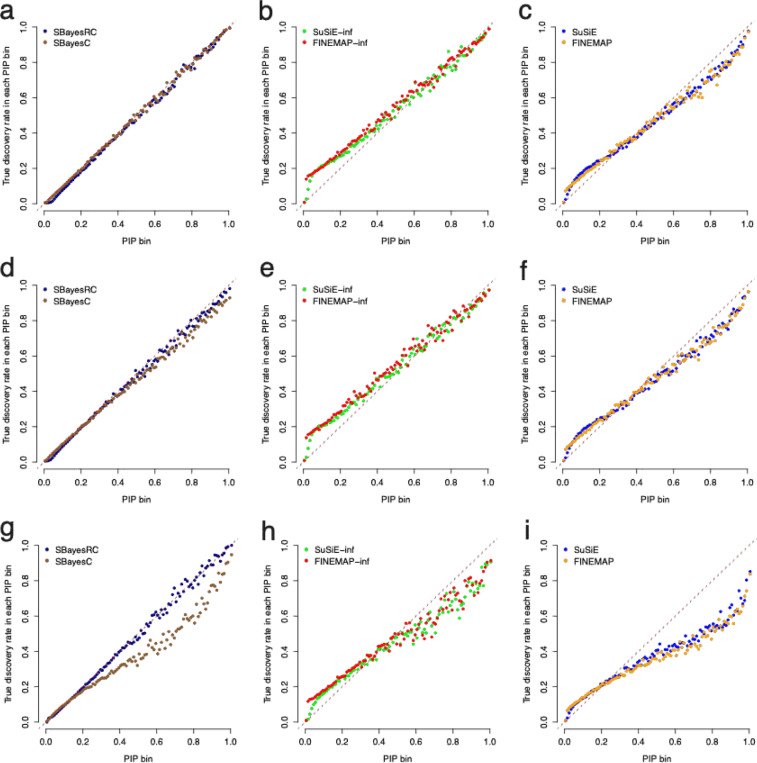
Comparison in the calibration of PIP between GBMM and existing fine-mapping methods under simulations with various genetic architectures. Shown are relationship between PIP and the true discovery rate across 100 PIP bins. Results showed in each column correspond to the results from GBMM (SBayesC and SBayesRC), SuSiE-inf and FINEMAP-inf and SuSiE and FINEMAMP respectively. Results shown in each row correspond to the sparse genetic architecture, major gene genetic architecture and LDMS architecture respectively.

Results showed that overall, the GWFM methods had the best calibration, the enhanced region-specific methods with an infinitesimal effect (i.e., FINEMAP-inf and SuSiE-inf) the second, and the standard region-specific methods (i.e., FINEMAP and SuSiE) the worst (Fig. 2). Under the sparse genetic architecture, PIPs from SBayesRC/SBayesC were in strong concordance with the TDR across its full spectrum (Fig. 2a). The concordance was reasonably good for SuSiE-inf and FINEMAP-inf, although with a trend of deflation in SNPs with low PIP, whereas for SuSiE and FINEMAP, even in SNPs with high PIPs, a notable inflation was observed, indicating a lack of control of FDR (=1-TDR) (Fig. 2b-c). When the large-effects or LDMS architecture was used, the assumption in the point-normal BMM was violated in a way that the causal effects did not come from a single normal distribution or that the causal variants were not randomly distributed across the genome. Consequently, PIPs from the point-normal BMM were no longer accurately tracking the true probabilities of causality. When the LDMS architecture was used, the FDR was even more poorly controlled in these methods (Fig. 2d-i). However, when SBayesRC was used, with LD and MAF bins as annotations, the strong concordance between PIP and TDR held in various architectures, although none of these architectures matched exactly with the analytic model assumed in SBayesRC.

In conclusion, the region-specific fine-mapping methods tended to have inflated FDR when the model assumptions were not met. In contrast, SBayesRC produced robust PIPs that were well calibrated under various genetic architectures.

### Local and global credible sets

**Figure 3:**
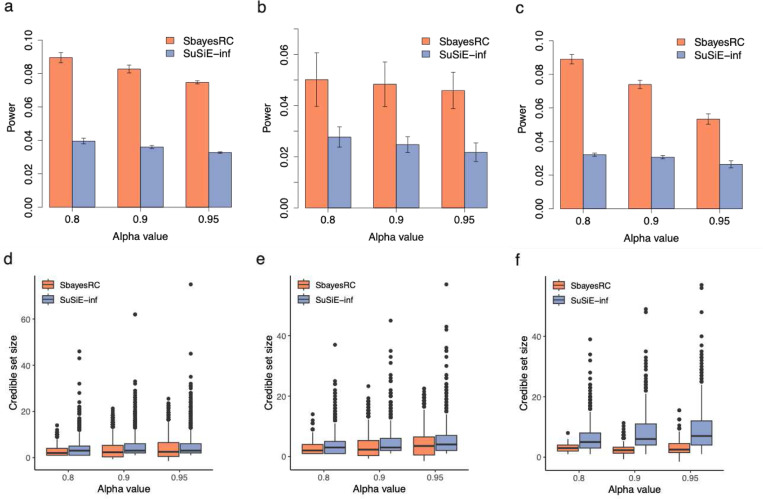
Comparison in local credible set (LCS) between SBayesRC and SuSiE-inf. Shown in panels (a-c) are power comparison between SBayesRC and SuSiE-inf at the same alpha cutoff. Shown in panels (d-f) are credible size comparison between SBayesRC and SuSiE-inf at the same alpha cutoff. Results showed in each column correspond to the simulation under sparse model (a, and d), major gene model (b and e) and LDMS model (c and f).

In addition to individual SNP PIP, CS is another critical statistic in fine-mapping. Here, we assessed the performance of SBayesRC in identifying LCS and GCS. For each LD block, we computed an α-LCS that contains at least a causal variant with a probability of α and is enriched in $h_{SNP}^{2}$ (PEP > 0.7). We first evaluated the true discovery rate for identifying LCS, defined as the actual fraction of the LCS with at least a causal variant. The simulation result showed that the SBayesRC has a similar TDR to SuSiE-inf, which had the best PIP calibration among the region-specific fine-mapping methods (**Fig. S1a-c**). However, SBayesRC was significantly more powerful (Fig. 3a-c) and had a remarkably smaller LCS size than SuSiE-inf at the same α threshold (Fig. 3d-f). For instance, when α=0.9, SBayesRC outperformed SuSiE-inf by up to 221% improvement in power and 41% reduction in LCS size across the three genetic architectures.

The α-GCS is expected to cover α proportion of the causal variants across the genome (Methods). Under various simulation scenarios, our GCS accurately represented the true proportion of causal variants (**Fig. S2a-c**), in contrast to the significant deflation observed with SBayesC (**Fig. S2a-c**). Furthermore, we observe a good agreement between estimated and observed power at any given PIP threshold from SBayesRC (**Fig. S2d-f**). Additionally, SBayesRC gave an unbiased estimate for the proportion of $h_{SNP}^{2}$ explained by the GCS SNPs, regardless of the given α value, under various scenarios (**Fig. S2g-i**).

### Improved mapping precision for identifying causal variants

**Figure 4:**
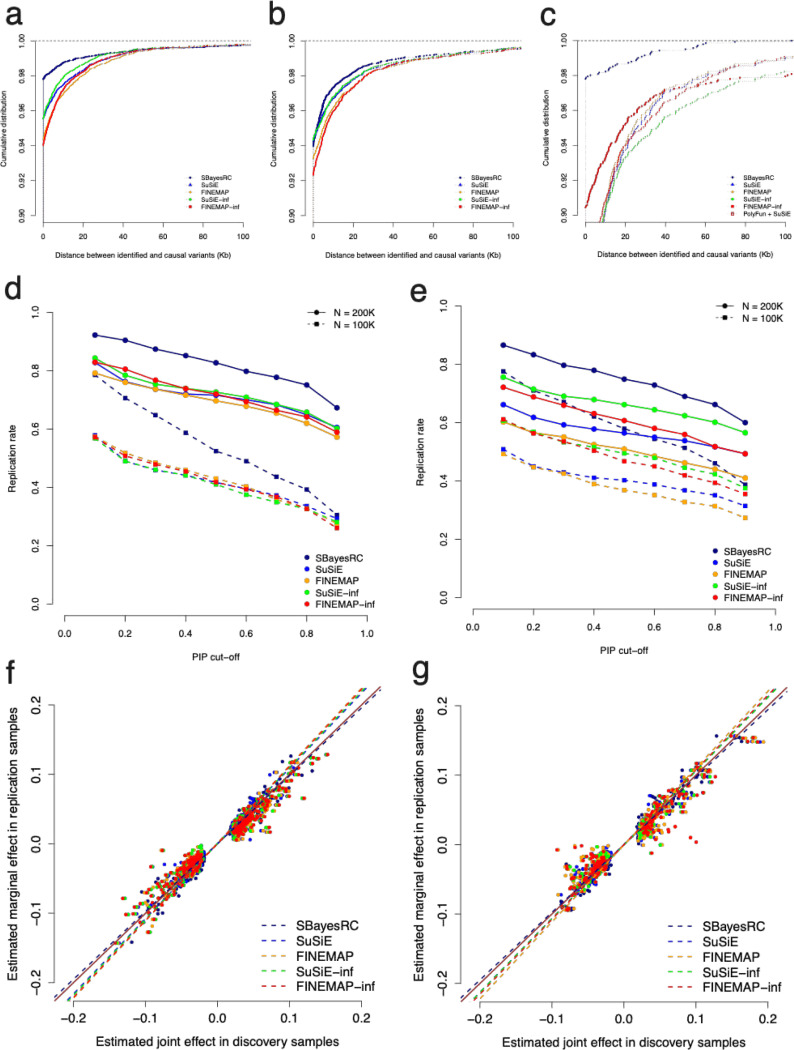
Comparisons of mapping precision, independent sample replication, and effect size estimation bias among fine-mapping methods. Panel (a-c) shows the distance between the causal variants and the SNPs identified by different methods at PIP of 0.9 in simulations based on sparse (a), large effects (b) and LDMS (c) genetic architectures (Methods). Panel (d-e) show the replication rate of discovery using different methods at a given PIP threshold in the replication sample (x-axis) using simulations (d) and real data analysis for height in the UKB (e). Simulations are based on a sparse model and results in (d) are the mean values over ten simulation replicates. Panel (f-g) show the regression of the estimated marginal effect size in replication samples on the estimated joint effect size in discovery samples using different fine-mapping methods. Dash line shows the regression slope, which is closer to one for a less biased method. The marginal effect estimated in the independent replication samples was used as a proxy to the true value because it is an unbiased estimate. The brown solid line is the y=x line.

Our simulation results have shown that SBayesRC had the best calibration even under the architecture that matched with the assumed model for the region-specific fine-mapping methods. We next quantified the mapping precision of these methods. The mapping precision was defined as the distance of the identified variant that passed a given PIP threshold to the nearest causal variant. Hence, the smaller the distance, the higher the mapping precision, e.g., the distance is zero if the causal variant itself is identified. Results from the sparse architecture simulation showed that 97.8% of SBayesRC identified SNPs with PIP > 0.9 were the causal variants, and 99% significant SNPs were located within 16.4kb distance to the causal variants (Fig. 4a). With the same PIP threshold, 95.5% and 94.3% of SuSiE-inf and FINEMAP-inf identified SNPs were the causal variants, slightly higher than that of 95.3% and 94.0% from SuSiE and FINEMAP, with 99% significant SNPs located within 25.8kb (SuSiE-inf) and 31.3kb (FINEMAP-inf) to the causal variants, compared to that of 32.7kb and 36.7kb for SuSiE and FINEMAP, respectively. In conclusion, given a PIP threshold of 0.9, SBayesRC led to an at least 2% increase in TDR and a 64% (16.4kb/25.8kb) reduction in the distance to the causal variants, both indicating improved mapping precision compared to the existing methods. We also ran a LD block-wise SBayesC analysis, with model parameters estimated from each region separately. Our result showed that the mapping precision remained notable higher than the competing region-specific fine-mapping methods (**Fig. S3**).

In the simulation with large-effects architecture, the mapping precision for all methods decreased due to the decrease of average per-SNP heritability (from 0.5/10,000 to 0.4/9,990). However, SBayesRC still had the highest precision among all methods (Fig. 4b). In the simulation with LDMS architecture, SBayesRC demonstrated a substantially higher mapping precision than the other methods (Fig. 4c), likely because SBayesRC allowed the model to weigh SNPs differentially based on their LD and MAF property so that the causal variants were better identified. Furthermore, we compared SBayesRC to Polyfun+SuSiE^19^, which is a stepwise method that accounts for the effect size stratification (by LD and MAF annotations) through a prior estimated from stratified LD score regression (S-LDSC)^37,38^. Indeed, Polyfun+SuSiE improved the mapping precision compared to the region-specific methods, but was still significantly inferior to SBayesRC (Fig. 4c). These simulation results suggested that SBayesRC is a reliable method for GWFM and can substantially improve the mapping precision of identifying causal variants.

### Improved replication rate of identification with less bias in estimation

In real data analysis, direct evaluation of mapping precision is not feasible, because which variants have causal effects on a trait are often unknown. Alternatively, we can evaluate the replication rate of the identified variants using an independent sample^13^. Here, we define the replication rate as the proportion of variants with a significant PIP (e.g., PIP > 0.9) from the GWAS sample to be repeatedly identified in an independent (replication) sample with the same or a smaller PIP threshold. It is expected that the method that identifies most causal variants from the GWAS sample will have the highest replication rate, as the false positives are unlikely to be replicated.

We performed simulations using the UKB samples of European ancestry and split samples into independent datasets for discovery and replication. Putative causal variants were identified at the PIP threshold of 0.9 in the GWAS data (*n*=100,000). We then quantified the replication rate of the putative causal variants at different significance thresholds in two replication datasets (*n*=100,000 and 200,000). Using SBayesRC, roughly 33% of identified SNPs can be replicated at PIP > 0.9 when replication *n* = 100,000, and the replication rate increased to 71% when the replication sample size was doubled (Fig. 4d). It may seem counter-intuitive that only a fraction of SNPs was replicated despite using the same PIP threshold of 0.9 in both the discovery and replication datasets. This discrepancy is because there exists a sampling variation in the causal variants identified from distinct samples. As expected, the replication rate increased when using a lower threshold for replication, e.g., with PIP > 0.1, 79.6% of the identified SNPs can be replicated when replication *n*=100,000. Compared to other methods, SBayesRC demonstrated significantly higher replication rate at each of the PIP thresholds, while differences among the other four methods were small. We also quantified the replication rate in the reverse case where the GWAS sample size was 200,000 but the replication sample size was only 100,000, to mimic the reality that the sample size of replication data is often much smaller than that of discovery. In this scenario, we found that 19% of the identified SNPs can be replicated at PIP > 0.9 using SBayesRC, and the replication rate of SBayesRC remains significantly higher than that of other methods at each PIP threshold (**Fig. S4a**).

We then assessed the replication rate in the UKB height by constructing different discovery and replication datasets as in the simulation. The results were consistent with the observations from the simulation study (Fig. 4e and **Fig. S4b**). Compared to the region-specific methods, SBayesRC improved the replication rate by 11.3% (compared to FINEMAP) and by 1.2% (compared to SuSiE-inf) at PIP > 0.9 when replication *n*=100,000, and improved the replication rate by 19% (compared to FINEMAP) and by 3.5% (compared to SuSiE-inf) when replication sample size was doubled.

Moreover, we examined the bias in effect size estimates of putative causal variants identified from fine-mapping, through regressing their marginal effect sizes estimated from the replication samples on the joint effect sizes estimated from the GWAS sample (the regression slope is expected to be one for an unbiased estimation). In the simulation and UKB height analyses, the regression slope from SBayesRC was 0.978 and 0.974, respectively, superior to all the other methods (Fig. 4f-g), likely due to the genetic architecture was estimated simultaneously in SBayesRC but was preset or estimated locally in other fine-mapping methods.

These analyses suggested that the identified SNPs from SBayesRC are more likely to be causal because of the relatively high replication rate in independent samples and the negligible bias in effect size estimation, compared to the other methods.

### Improved prediction accuracy using fine-mapped variants

**Figure 5:**
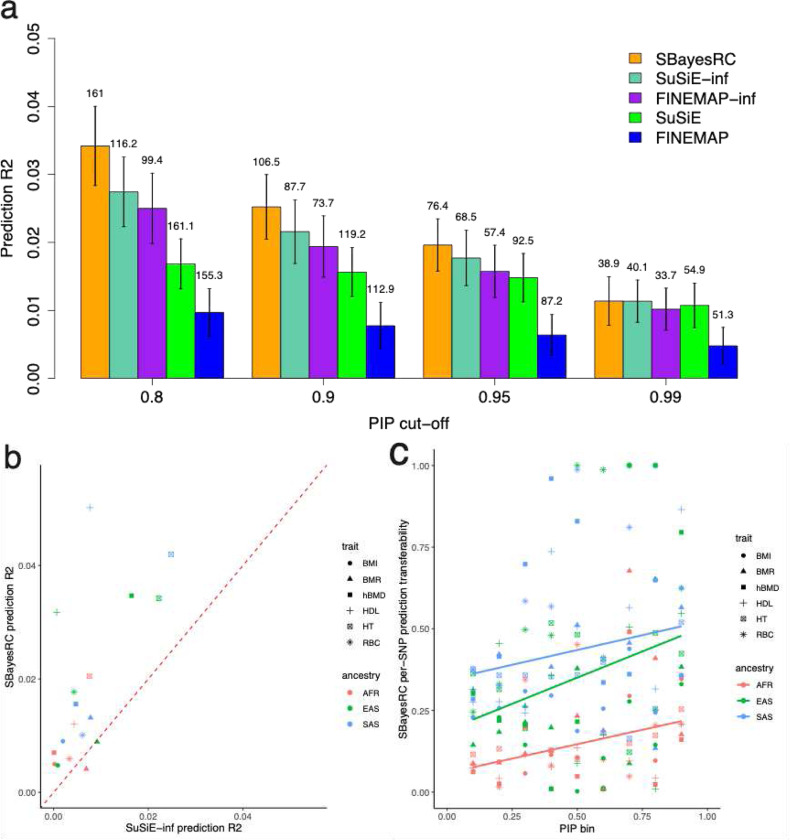
Out-of-sample prediction accuracy using identified variants from different fine-mapping methods. (a) Comparison of the prediction R2 using the fine-mapped SNPs from different methods in the simulation based on the sparse architecture (Methods). The number above each bar is the number of fine-mapped SNPs from each method at different PIP cut-offs. (b) Comparison of trans-ancestry prediction accuracy using fine-mapped variants from SBayesRC and SuSiE-inf from the analysis of samples of European ancestry for 6 complex traits in the UK Biobank, with variants with PIP > 0.9. (c) The relationship between trans-ancestry prediction transferability and PIP in European ancestry. The transferability was computed as non-EUR-R^2^/ EUR-R^2^. The solid lines are regression lines across traits in each ancestry. Results are the mean values over 100 simulation replicates.

Another approach to evaluate the results of fine-mapping is to conduct an out-of-sample prediction using the fine-mapped variants. Since all the Bayesian methods used in this study provide the posterior mean of SNP effects, we computed polygenic scores (PGS) based on the identified variants from each of the methods and evaluated the prediction accuracy in a validation sample. We split the 100K samples into 95K training samples to perform the fine-mapping analysis using all these Bayesian methods and predicted the phenotype for 5K independent individuals as validation samples. We found that overall, SBayesRC had a higher prediction accuracy compared to the other methods, outperforming them by at least ~17% at a PIP threshold of 0.9, with a relatively smaller number of SNPs included in the predictor (Fig. 5a). This is consistent with the result that SBayesRC resulted in a lower FDR than the other methods (Fig. 2a).

We further compared the performance of these methods by trans-ancestry prediction in real traits. Specifically, we used the fine-mapped variants and estimated posterior effect sizes obtained from the UKB individuals of the European (EUR) ancestry to predict the phenotypes in three other ancestries in UKB: African (AFR), East Asian (EAS) and South Asian (SAS). We selected 6 complex traits that had at least 50 identified SNPs at a PIP threshold of 0.9. We compared the performance of trans-ancestry prediction between SBayesRC and SuSiE-inf, because SuSiE-inf has exhibited a superior performance compared to the others. The result showed that compared to SuSiE-inf, SBayesRC improved the trans-ancestry prediction accuracy using fine-mapped variants, with a nearly 10-fold increase in the mean relative prediction ${\text{R}}^{2}\left(\frac{R_{\text{SBayesRC}}^{2}-R_{\text{SuSiE-inf}}^{2}}{R_{\text{SuSiE-inf}}^{2}}\right)$ across traits and ancestries (Fig. 5b). We further compared the performance of SBayesRC and SuSiE-inf for trans-ancestry prediction using the identified credible set SNPs. Similar to the comparison result based on fine-mapped variants, SBayesRC improved the trans-ancestry prediction accuracy based on the SNPs in the 90-LCS (**Fig. S5**; 1.7-fold increase on average). Since it is parsimonious to assume that the common causal variants and their effect sizes are mostly shared between ancestries^39,40^, we expect to observe a strong concordance in prediction accuracy between EUR and other ancestries using putative causal variants identified from the EUR sample with high confidence. To investigate this, we quantified the transferability of fine-mapped SNPs by computing the ratio of per-SNP prediction accuracy in a hold-out EUR sample to that in a different ancestry. The result showed that on average this relative prediction accuracy of SNP increases with its PIP calculated in the EUR GWAS sample (Fig. 5c). These results suggested that SBayesRC has higher power of fine-mapping and higher accuracy of variant effect estimation.

### Prediction of fine-mapping power and variance explained

**Figure 6:**
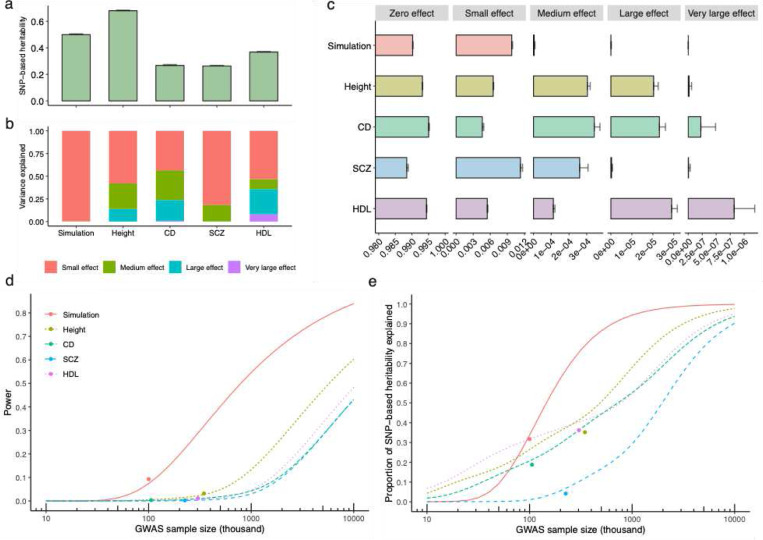
Projection of genome-wide fine-mapping outcomes to the theoretical power prediction in complex traits with diverse genetic architectures. (a-c) show the results of SBayesRC estimation for the SNP-based heritability $\left(h_{SNP}^{2}\right)$ (a), the proportions of $h_{SNP}^{2}$ explained by different mixture components (b), and the proportions of SNPs with effects from different mixture components (π) (c), for the simulated trait, height, Crohn’s disease (CD), schizophrenia (SCZ), and high density lipoprotein (HDL). (d-e) shows the theoretical prediction of the power of identifying causal variants (d) and the proportion of $h_{SNP}^{2}$ explained by the identified causal variants at different GWAS sample sizes for these traits. Dot shows the observed trait outcome based on local credible sets (including singleton LCSs) identified from SBayesRC. Note that the results shown in (a, c) were used as input data for our method that predicts fine-mapping power given sample sizes (d-e).

As a unique feature of the GWFM approach, the genetic architecture estimated from SBayesRC provides information to predict the proportion of causal variants identified from fine-mapping (power) and the proportion of $h_{SNP}^{2}$ explained by these variants (PHE) for future studies (Methods and **Supplementary Note**). To evaluate our approach, we computed the predicted values of power and PHE at a spectrum of GWAS sample sizes and projected the outcome of fine-mapping using SBayesRC onto the prediction using data from the simulated trait with sparse architecture, height^36^, high density lipoprotein (HDL), schizophrenia (SCZ)^41^, and Crohn’s Disease (CD)^42^. These traits were selected to represent different genetic architectures (Fig. 6a-c). Despite some variations across traits, the outcomes from the fine-mapping analyses were overall consistent with the theoretical predictions (Fig. 6d,e). While our theoretical prediction does not model LD between SNPs, the extent to which the observed values were consistent with the predicted suggests that LD had been effectively, albeit not perfectly, accounted for by our LCSs.

Take SCZ for example. Using the latest GWAS summary statistics from the psychiatric genomics consortium dataset^41^, we identified 13 SNPs and 222 credible sets, collectively explaining 3.9% of $h_{SNP}^{2}$ at the liability scale^43^. These estimates are highly consistent with our theoretical prediction given the 53,386 cases and 77,258 controls in their study^41^, which is equivalent to a sample size of 228,810 on the liability scale (ref^44^; Methods). For a prospective study using SBayesRC, we predict that ~180k cases would be required to fine-map 1,000 common causal variants (estimated to be 1.2% of all common causal variants), assuming an equal number of controls and a population prevalence of 0.01 (Methods), collectively accounting for about 20% of $h_{SNP}^{2}$ (Fig. 6). With ~550k cases under the same assumption, we will be able to identify 10% causal variants explaining 50% of $h_{SNP}^{2}$ in SCZ. To fine-map variants accounting for 80% of $h_{SNP}^{2}$, it was estimated to require 1.4 million cases.

### Genome-wide fine-mapping in complex traits from UK Biobank

We applied GWFM with SBayesRC to 600 complex traits (598 from the UKB) and developed an online resource to query these fine-mapping results (see URLs; **Supplementary Table 3**). We selected these 598 UKB traits based on z-score > 4 and high confidence for heritability estimates using LD score regression (https://zenodo.org/records/7186871). To better capture the causal variants, we used 13 million imputed SNPs with functional genomic annotations from Finucane et al.^37^. Here, we focus on discussing the results for 48 complex traits that had sufficient power, including SCZ, CD^41,42^ and 46 UKB traits measured in the European ancestry inferred individuals (Methods). At the PIP significance threshold of 0.9, we identified 2,868 SNPs associated with 48 complex traits, 1,641 of which were not identified by LD clumping, and 22,803 0.9-LCSs with an average size of 8.7 SNPs. On average across these 48 traits, we estimated that although these fine-mapped variants and LCSs only captured 0.75% of the causal variants, they accounted for 17.4% of the $h_{SNP}^{2}$.

**Figure 7:**
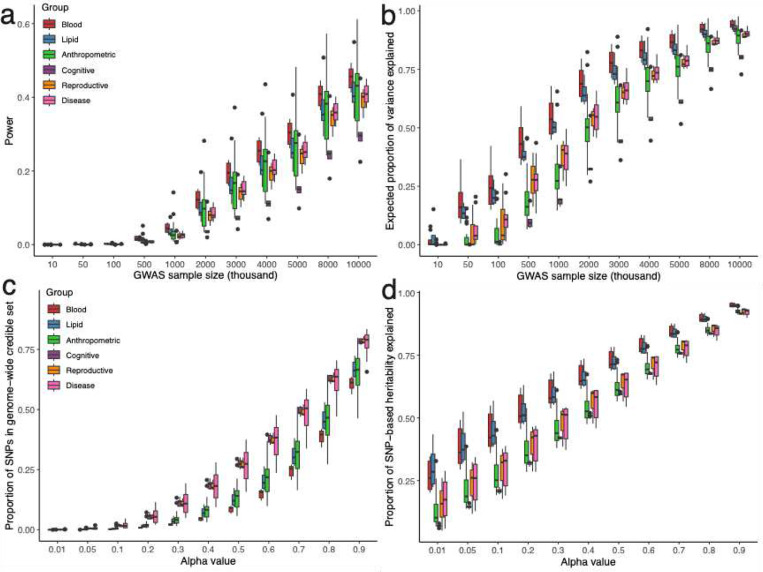
Theoretical identification and prediction of genome-wide credible SNPs across 48 independent complex traits. Panel (a-b) shows the theoretical prediction of power and proportion of SNP-based heritability explained by GCS SNPs at different GWAS sample sizes for the 48 complex traits, respectively. Panel (c) shows the proportion of identified GCS SNPs at different alpha threshold (proportion of causal variants captured) for the 48 complex traits (average sample size = 291K). Panel (d) shows the proportion of $h_{SNP}^{2}$ explained by the GCS SNPs at different alpha threshold. Colours indicate different trait categories.

Given the estimated genetic architecture for these 48 traits, we applied our theoretical prediction approach to predict the power of prospective GWAS studies. With a GWAS sample size of 2 million individuals, we predict that the average power is 9.5% (Fig. 7a) and average PHE is 54.1% (Fig. 7b). The predicted values varied substantially between trait groups. Blood cell traits had both the highest power (29.7%) and highest expected proportion of $h_{SNP}^{2}$ explained (86.9%), while cognitive traits have the smallest power (16.9%) and smallest expected proportion of genetic variance explained (63.1%). To achieve a PHE of 50%, blood cell traits require a GWAS sample size of only 1 million individuals, while cognitive traits necessitate a sample size of 5 million individuals, due to the differences in genetic architecture across complex traits. The required sample size increased to 3 million and 10 million for blood cell counts and cognitive traits, respectively, to achieve a PHE of 80% (Fig. 7b).

The global credible set α-GCS varied in the credible set size and PHE estimate across traits (Fig. 7c). On average, the 0.1-GCS, i.e., covering 10% of causal variants, consisted of 1% of the genome-wide SNPs, which explained 31.8% of the $h_{SNP}^{2}$ (Fig. 7d). Among the analysed complex traits, diseases had the largest GCS sets, requiring 1.9% genome-wide common SNPs to cover 10% common causal variants. In contrast, blood cell traits had the smallest GCS, requiring merely 0.21% genome-wide common SNPs to cover 10% common causal variants. Interestingly, the 0.1-GCS for blood cell traits explained 44.3% of the total genetic variance, compared to 20.8% explained by the GCS for cognitive traits, highlighting the less polygenic genetic architecture of blood cell traits.

**Figure 8:**
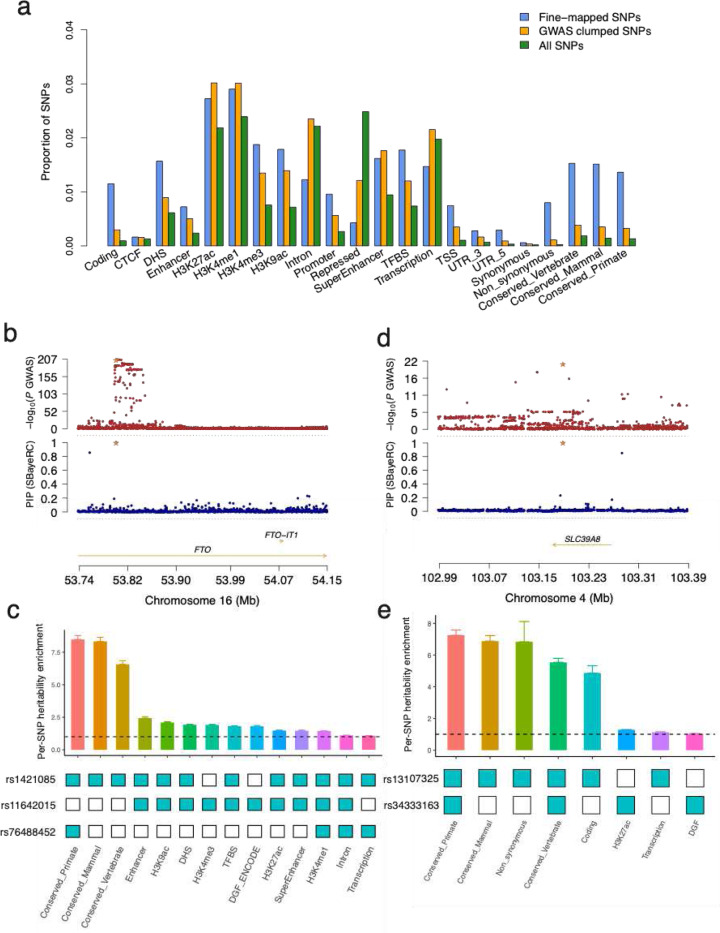
Genome-wide fine-mapping with functional annotations helped pinpoint the putative causal variants. Panel (a) shows enrichment of the genome-wide fine-mapped SNPs from SBayesRC and GWAS clumped SNPs in the 22 main functional categories defined in the LDSC baseline model. Panel (b) shows the prioritized causal variant at the *FTO* locus for BMI. The top track shows the *FTO* locus plot of the standard GWAS for BMI, and the second track shows the similar plot but with the PIP from SBayesRC for BMI. The starred SNP is the known causal variant (rs1421085) for obesity at the *FTO* locus supported by previous functional studies. Panel (c) shows the per-SNP heritability enrichment for the causal variant (rs1421085), the GWAS lead variant (rs11642015) and the secondary signal (rs76488452) for BMI at the *FTO* locus. The annotations on the x-axis were those present at least once in these three variants, excluding quantitative annotations and duplicated annotations with flanking windows. Panel (d) shows the prioritized causal variant at the *SLC39A8* locus for SCZ. The top track shows the *SLC39A8* locus plot of the standard GWAS for SCZ, and the second track shows the similar plot but with the PIP from SBayesRC for SCZ. The starred SNP is the missense variant (rs13107325) fine-mapped for SCZ at the *SLC39A8* locus. Panel (e) shows the per-SNP heritability enrichment for the causal variant (rs13107325) and the secondary signal (rs34333163) for SCZ at the *SLC39A8* locus.

Over the 48 complex traits, the number of fine-mapped variants from SBayesRC was highly correlated with the number of identified GWAS loci (**Fig. S6**), ranging from 1 to 489, with an average of 86.2 across complex traits (**Fig. S7**). Compared to the genome-wide SNPs and GWAS identified SNPs after LD clumping, the 2,868 putative causal variants had a significant overrepresentation in functional genomic regions included in the functional genomic annotations^37^, such as coding, promoter, and enhancer regions, and were significantly depleted in repressed regions (Fig. 8a), suggesting the importance of functional annotations. Of these variants, 651 (22.8%) were in association with more than one complex trait, highlighting the prevalence of pleiotropy in human genome. Moreover, the number of traits that the variant had pleiotropic effects decreased with its minor allele frequency (**Fig. S8**), consistent with that highly pleiotropic variants would be expected to be removed from the population or kept at low frequencies due to natural selection^45^.

### Functional annotations helped pinpoint the putative causal variants

One notable example is a variant (rs1421085) at the FTO locus, which was identified to be a putative causal variant using SBayesRC for body mass index (BMI), body fat percentage (BFP), hip circumference (HC) and waist circumference (WC). It has been previously validated that this variant plays a causal role in adipocyte thermogenesis regulation^46^. Unlike the results from the standard GWAS where many SNPs in the FTO locus exhibited a signal at genome-wide significance level, our analysis showed that only the known causal variant (rs1421085) was significantly (PIP > 0.9) associated with BMI (Fig. 8b). In contrast, applying other methods (SBayesR and SuSiE-inf) without functional annotations to the locus identified the GWAS lead SNP instead, underscoring the importance of incorporating functional annotations. In particular, the annotations of conservation across species, especially primates, helped distinguish the causal variant from the GWAS lead SNP (Fig. 8c). Moreover, a secondary signal rs76488452 (PIP=0.85) was identified by SBayesRC, which has not been previously reported but was included in a local credible set of 5 SNPs in both SBayesR and SuSiE-inf (**Fig. S9**). We found this SNP resided in a conserved region in primates and was also significant in the COJO analysis (p-value = 1.8×10^−17^) conditional on the known causal variant. Notably, the secondary signal (rs76488452) was only nominally significantly in the GWAS marginal analysis (p-value = 3.6×10^−4^), whose trait-increasing allele was in negative LD (*r*=−0.16) with that of the known causal variant, indicating that this SNP is likely to have a masked effect^47^ (estimated masked effect size $b_{2}-r*b_{1}=0.02$, consistent with the reported GWAS marginal effect size).

Another example is from the fine-mapping results for SCZ. We identified 13 SNPs at PIP > 0.9 for SCZ from the latest meta-analysis, 5 of which were the same SNPs that were identified using FINEMAP in their study^41^, and all the 8 SNPs identified by FINEMAP were included in our 0.9-LCSs. We recapitulated a missense variant (rs13107325) in *SLC39A8*, a gene highlighted in the latest SCZ analysis for its function in regulating dendritic spine density^48,49^. Furthermore, we identified a secondary variant at the same locus, located in important functional regions (Fig. 8d-e). Among the 5 novel fine-mapped SNPs that were not identified by FINEMAP with individual PIP, 3 were missense variants (**Fig. S10a-c**). We highlight rs11845184, which is located within *SECISBP2L* (**Fig. S10a**). *SECISBP2L* is highly expressed in brain-related tissues (**Fig. S11**), specifically in differentiating oligodendrocytes, where the SECISBP2L-DIO2-T3 pathway mediates the autonomous regulation of oligodendrocyte differentiation during myelin development^50^. Moreover, we identified novel putative causal variants for CD (**Supplementary Table 4**). Using SBayesRC, we fine-mapped 31 variants, of which 10 were missense variants, and all 3 variants identified in the previous study using the same data were recapitulated^42^. In addition, compared to a recent exome-wide association study for CD^51^, we identified 4 novel genes (*LACC1*, *SLAMF8*, *MAN2B2* and *GPR35*) with missense putative causal variants (**Fig. S12**). These results demonstrated the power of SBayesRC for identifying the plausible causal variants and provide a valuable resource for downstream analysis and functional validation.

## Discussion

In this study, we comprehensively evaluated the performance of GWFM using SBayesRC by extensive simulation and real data analyses, compared with the existing fine-mapping methods that consider one genomic region at a time. Our results showed that both PIP and CS from SBayesRC were correctly calibrated under various genetic architectures, indicating well controlled FDR. In contrast, the other methods produced mis-calibrated PIP and CS with inflated FDR, when the genetic architecture did not match the model assumption. While all fine-mapping methods gave a higher mapping precision than that from GWAS^7^, SBayesRC had the highest precision across genetic architecture scenarios. Furthermore, in both simulation and real trait analyses, SBayesRC showed significantly higher replication rate and prediction accuracy but less estimation bias in an independent sample using fine-mapped SNPs, compared with the other methods. In the real data analysis, we showed examples where SBayesRC pinpointed the putative causal variants that were missed by the other methods. All of these results indicate that SBayesRC, as a method for GWFM analysis, remarkably improves the identification of causal variants.

We proposed a new LD-based method to compute LCSs and estimate their contribution to the SNP-based heritability. This method overcomes the limitation of existing window-based approaches that causal variants with SNPs in long-range LD would not be captured. In addition to LCSs, SBayesRC allows us to compute a GCS for the trait, which informed the power of identifying the causal variants and the $h_{SNP}^{2}$ explained by the identified SNPs given the data. This computation requires an unbiased estimation on the total number of the causal variants, which can only be done when analysing all SNPs jointly in the model. The analysis of 48 complex traits showed that although as many as 22,803 variants or LCS were identified, they only captured 0.75% of all common causal variants and contributed 17.4% genetic variance, suggesting many causal variants with very small effects are yet to be discovered (Fig. 7c,d).

We have provided a theoretical prediction of fine-mapping power given a sample size and the estimated genetic architecture (**Supplementary Note**). This is useful to inform the experimental design of future fine-mapping studies regarding the sample size required to identify a certain number of causal variants or those explaining a certain proportion of $h_{SNP}^{2}$. The robustness of our prediction approach is supported by projecting the outcomes of real data analyses to the landscape of predicted values. For height, based on the UKB data (n = 350k), we predicted that when the sample size increases to 5 million, the number of fine-mapping discoveries would be ~10,000 considering significant PIPs only or ~30,000 considering both significant PIPs and LCSs, explaining up to 95% of the genetic variance (Fig. 6). This prediction is consistent with the finding of a recent GWAS with 5 million individuals, which reported 12,111 independently significant SNPs identified from COJO accounting for nearly all of the common SNP-based heritability in height^40^.

While the concept of credible set has evolved over time^11,12,52^, it is still common to focus on individual SNP PIP in the downstream analysis, probably because the CS include too many SNPs to follow up. Our study provided important implications regarding this issue. First, CS may miss the true causal variant if not all possible causal variants are fitted in the model, underscoring the importance of considering all common SNPs in the fine-mapping analysis. Second, our GWFM approach can reduce the credible size, as shown in both simulation and real trait analysis (only ~8.7 SNPs per credible set), facilitating the consideration of CS in practice. Third, in the presence of complete LD between SNPs and the causal variants, the PIP of a causal variant may never be significant regardless of sample size, but leveraging functional genomic annotations may help distinguish causal from non-causal variants. In this sense, genomic annotations play a greater role than the increase of GWAS sample size.

The advantages of SBayesRC over the region-specific fine-mapping methods arise from the following aspects. First, SBayesRC involves a genome-wide analysis fitting all SNPs jointly. Compared to the region-specific analysis, genome-wide analysis accounts for long-range LD and utilises all SNPs to estimate the genetic architecture, thereby improving fine-mapping. Of note, even when the same LD blocks are used in both types of analyses, the latter is still superior because of the better estimation of genetic architecture parameters from genome-wide SNPs. Second, SBayesRC assumes a more realistic distribution of SNP effects through using MAF/LD groups along with other functional annotations. In addition, the impact of annotations on the SNP effect distribution is estimated within the same model, fostering a formal Bayesian learning process. The existing fine-mapping methods, however, either do not leverage annotation data or lack a unified framework for the joint analysis with GWAS data. Third, SBayesRC utilises MCMC sampling to estimate model parameters and PIPs, which is asymptotically exact. Both FINEMAP and FINEMAP-inf use shotgun stochastic search, while both SuSiE and SuSiE-inf use variational Bayes to compute the Bayes factors for the causal models and therefore the PIPs. It has been previously shown that MCMC sampling generally leads to a higher accuracy of capturing the posterior distribution than the other approximation approaches^30^. To further justify our choice of SBayesRC as the method for GWFM, we ran SBayesRC within each block separately and quantified the mapping precision. We found that the mapping precision decreased compared to the genome-wide SBayesRC but remained higher than the other methods (**Fig. S2**). For example, 99% of SNPs identified by the region-specific SBayesRC were located within 23.1Kb to causal variants, compared to the number of 16.4Kb for the genome-wide SBayesRC and 25.8Kb for SuSiE-inf.

We note several limitations of this work. First, there are certainly more complicated scenarios about effect size distribution for causal variants that have not been investigated in our simulations. However, to our knowledge, SBayesRC is one of the most flexible models to accommodate various scenarios because it assumes a multi-component Gaussian mixture, and we have further allowed the method to automatically choose the number of mixture components. Second, unlike an individual-level model where each PIP is calculated conditional on the effects from all other SNPs jointly, SBayesRC is a summary-level model where LD between LD blocks is ignored so that SNPs beyond the region contribute to the PIP only through the mixture distribution of SNP effects. Third, we only applied our method to the GWAS summary data from relatively homogenous populations (inferred European ancestry) and the robustness of the methods on GWAS data based on trans-ancestry meta-analyses is not investigated. Fourth, SBayesRC requires the LD information estimated from a cohort that matches with GWAS ancestry without substantial sampling variation. Fifth, to create the credible set, a threshold of 0.5 was arbitrarily chosen to define a set of SNPs in high LD. Latest methodological advancements in Bayesian hypothesis tests based on hierarchical clustering can be used to relax this condition^53^. Sixth, the prediction of mapping power is based on the genetic architecture estimates given a SNP set. However, the SNP set may change with the sample size (e.g., more common SNPs to be observed in a larger sample size), which may affect the polygenicity and SNP-based heritability. Despite these limitations, our study provides a robust and versatile GWFM framework for identifying causal variants, highlighting the advantages of this approach over existing region-specific fine-mapping methods. With its capacity to enhance mapping power in the current study and to predict mapping power for future studies, we believe GWFM using a state-of-the-art GBMM will become the preferred method for analysing complex traits.

## Methods

### Low-rank GBMM

We used state-of-the-art GBMM that employed a low-rank model to improve computational efficiency and robustness^27^. As described below, the low-rank GBMM can be derived from the individual-level model. Consider a multiple linear regression of phenotypes on genotypes:

(1) y=Xβ+e

where y is an n×1 vector of complex trait phenotypes and X is an n×m matrix of mean-centred genotypes, β is m×1 vector of SNP effects on the trait, and e is n×1 vector of residuals with $\text{var}(e)=I\sigma_{e}^{2}$. Let

(2) $$K=\Lambda^{-\frac{1}{2}}U^{\prime }X^{\prime }n^{-1}$$

where Λ and U are diagonal matrix of eigenvalues and matrix of eigenvectors for the LD correlation matrix $R=X^{\prime }X/n$, respectively. It follows that $K^{\prime }K=Pn^{-1}$, where P is the projection matrix of y on the column space of X, and $KK^{\prime }=In^{-1}$. Multiplying both sides of Eq (2) by K gives

(3) Ky=KXβ+Ke

or

(4) w=Qβ+ϵ

When only the top q principal components of R are used, the dimension of w is q×1 and Q is q×m. Since q≪n, this model would have a substantially lower rank than Eq (1), improving the computational efficiency for the estimation of β. With a recognition of $b=X^{\prime }y/n$ is the GWAS marginal effect estimates, w can be directly computed from the GWAS summary statistics. In practice, we compute w and Q within quasi-independent LD blocks in the human genome. With this low-rank model, we can estimate β for all common variants jointly through considering β as random effects. In addition, this model allows a direction estimation of the residual variance, as $\text{var}(\epsilon )=I\sigma_{e}^{2}n^{-1}$, which can be used as a nuisance parameter to accommodate heterogeneity in the summary statistics and LD reference^27^.

### SBayesC and SBayesRC

GBMMs are flexible in the specification of the prior distribution of SNP effects. In SBayesC, the prior for the effect size of variant j is,

(5) $$\beta_{j}∼N\left(0,\sigma_{\beta }^{2}\right)\pi +\phi (1-\pi )$$

where $\sigma_{\beta }^{2}$ is the common variance across all the causal variants, π is the proportion of SNPs with nonzero effects, and ϕ is a point mass at zero. Both $\sigma_{\beta }^{2}$ and π are considered as unknown, with a scaled inverse chi-squared prior distribution and a uniform prior distribution, respectively^27^.

SBayesRC^27^ is an extension of SBayesR^24^, which allows for a more realistic prior distribution of SNP effects by assuming a multiple component mixture distribution

(6) $$\beta_{j}∼\sum_{k=1}^{5}\pi_{k}N\left(0,\gamma_{k}\sigma_{g}^{2}\right)$$

where $\gamma ={\left(\gamma_{1},\gamma_{2},\gamma_{3},\gamma_{4},\gamma_{5}\right)}^{\prime }={\left(0,1\times 10^{-5},1\times 10^{-4},1\times 10^{-3},1\times 10^{-2}\right)}^{\prime }$ are the prespecified coefficients to constrain the variance in each effect size distribution with respect to the total genetic variance $\left(\sigma_{g}^{2}\right)$, and $\pi_{k}$ is the prior probability for the SNP effect belong to the kth distribution. To further account for the stratification of causal variants and their effects regarding functional annotations, SBayesRC assumes a SNP-specific prior probability of distribution membership, $\pi_{jk}$, depending on the annotations at each SNP, through a generalised linear model. Specifically,

(7) $$f\left(\pi_{jk}\right)=\mu_{k}+\sum_{l=1}^{c}A_{jl}\alpha_{kl}$$

where f(⋅) is the probit link function, $\mu_{k}$ is the intercept, $A_{jl}$ is the value of annotation l on SNP j (either binary or continuous annotations), and $\alpha_{kl}$ is the effect of annotation l on the prior probability of the SNP effect belonging to the *k*th distribution. Details of the method can be found in ref^27^.

### Calculation of PIP

We assessed the strength of joint association of each SNP using the posterior inclusion probability (PIP), i.e., the probability of a SNP being included with a nonzero effect in the model, given the data. Let $\delta_{j}$ be the indicator variable for the distribution membership for SNP j, with $\delta_{j}=1$ indicating a null effect and $\delta_{j}=2,\ldots ,K$ indicating a nonnull component. We computed PIP for SNP j as

(8) $${\text{PIP}}_{j}=1-\mathrm{Pr}\left(\delta_{j}=1|y\right)=1-\frac{f\left(y|\delta_{j}=1\right)\pi_{1}}{\sum_{k=1}^{J}f\left(y|\delta_{j}=k\right)\pi_{k}}$$

The likelihood function when $\delta_{j}=1$ are

(9) $$f\left(y|\delta_{j}=1\right)\propto \exp\left\{-\frac{y_{c}^{\prime }y_{c}}{2\sigma_{e}^{2}}\right\}$$

where $y_{c}$ is the adjusted y for all other effects except that for SNP j.

The likelihood function when $\delta_{j}=k$ is

(10) $$f\left(y|\delta_{j}=k\right)\propto \exp\left\{-\frac{y_{c}^{\prime }y_{c}}{2\sigma_{e}^{2}}\right\}\lambda_{k}^{\frac{1}{2}}C_{k}^{-\frac{1}{2}}\exp\left\{\frac{r^{2}}{2c_{k}}\right\}$$

where $\lambda_{k}=\frac{\sigma_{e}^{2}}{\gamma_{k}\sigma_{g}^{2}},C_{k}=n+\lambda_{k},r=X^{\prime }y_{c}=X^{\prime }e+n\beta$. A full derivation of equation above can be found in the supplementary Note.

For all GBMM analyses in this study, we ran Markov chain Monte Carlo (MCMC) sampling for 10,000 iterations with the first 2,000 samples as burn-in and we used the posterior mean over 8,000 posterior samples to estimate π and PIPs.

### Automatic determination of mixture components

The standard SBayesRC requires specification of the number of mixture components before the analysis. It has been shown that the performance of polygenic prediction is robust to the number of mixture components^27^. However, this may be a problem for fine-mapping if a small effect component is unnecessarily included, where null SNPs are fitted by chance to explain negligible variance. This is because these SNPs may affect the distribution of PIPs and cause a bias in the estimation of the number of causal variants. In this study, we have allowed the method to automatically determine the number of mixture components for SBayesRC. The procedure started with running SBayesRC using the default setting of five mixture components. After 500 iterations of MCMC, the smallest component would be removed if the genetic variance explained by the SNPs in this component were less than half of that in the second smallest component. This procedure was repeated until no component was removed from the model. The rationale is that in most complex traits, due to the action of negative selection, most variation is attributed to variants with small effects^31,32,54^. Hence, if the smallest component is capturing true genetic effects, it should contribute to a significant proportion of variance, unlikely to be substantially lower than the second smallest component.

### Local and global credible sets

Similar to prior work^14^, we defined the local credible set SNPs as the minimum set of SNPs that contains at least one causal variant with a probability of α. To identify the α-LCS, we ranked SNPs based on their PIPs and constructed candidate credible set for each “free” SNP which was not in any LCSs. For the focal SNP, the candidate credible set was created by including “free” SNPs in high LD (r^2^ > 0.5) with a focal SNP and computed the α-LCS by summing over PIPs in a decreasing order until the sum is at least α. This process was iteratively repeated until all SNPs were exhausted. For each α-LCS, we calculated an LCS posterior SNP-heritability enrichment probability, where PEP is the probability that the focal LCS explains more $h_{SNP}^{2}$ than a random set of SNPs with the same size. We reported all the 0.9-LCS with PEP > 0.7 for each LD block. The true discovery rate was quantified as the proportion of identified α-LCS containing at least one causal variant, and the power was calculated as the proportion of simulated causal variants included in the identified α-LCS.

Analogous to the LCS, which identifies a set of SNPs that capture a causal variant with a probability of α, the GCS identifies a set of SNPs that capture all causal variant with a probability of α, which is equivalent to finding a set of SNPs that capture α% of the causal variants. We computed the α-GCS as the cumulative sum of decreasingly ranked PIPs that was greater than $\alpha \times m_{c}$, where $m_{c}$ was the estimated number of causal variants from GBMM for the trait. The α-GCS is expected to cover α% of all causal variants for the trait, i.e., the power of identifying the causal variants given the data (**Supplementary Note**).

### Estimation of power and variance explained given the data

For the identified SNPs using individual PIP or credible set, we estimated the power of identifying the causal variants given the data at a given threshold α,

(11) $${\text{TPR}}_{\alpha }=\frac{\sum_{j}\left[{\text{PIP}}_{j}|{\text{PIP}}_{j}\geq \alpha \right]}{M\pi }$$

A formal derivation is given in the **Supplementary Note**.

Moreover, we estimated the proportion of SNP-based heritability explained (PHE) by LCSs. Specifically, we computed PHE for a focal set (i) of SNPs in each MCMC iteration using the sampled values of SNP effects,

(12) $${\text{PHE}}_{mcmc_{r},i}=\frac{\sum_{j}\left[\beta_{mcmc_{r},j}^{2}|j\in i\right]}{\sum_{m=1}^{M}\left[\beta_{mcmc_{r},m}^{2}\right]}$$

where ${\text{β}}_{mcmc_{r},j}$ is the sampled effect for SNP j from MCMC iteration r in the scaled genotype unit. Finally, we computed the posterior mean across MCMC iterations as the estimate for ${\text{PHE}}_{i}$,

(13) $${\text{PHE}}_{i}=\frac{\sum_{r}{\text{PHE}}_{mcmc_{r},i}}{L}$$

where L is the total number of MCMC iterations.

### Prediction of power and variance explained for prospective studies

We aim to predict the power of identifying a certain proportion of the causal variants in a prospective fine-mapping analysis, given a sample size (n) and the genetic architecture of the trait, when PIP from a GBMM is used as the test statistic. As shown in the **Supplementary Note**, assuming that variance explained by the causal variant is v, the sampling distribution of PIP from the multi-component mixture model, e.g., SBayesRC, is

(14) $$PIP=1-\frac{1}{1+\sum_{k=2}^{5}A_{k}\exp\left\{B_{k}Z\right\}}$$

where $A_{k}=\frac{\pi_{k}}{\pi_{1}}\lambda_{k}^{\frac{1}{2}}C_{k}^{-\frac{1}{2}}$ and $B_{k}=\frac{n\sigma_{e}^{2}}{2C_{k}}$ are two constants given the genetic architecture parameters $\left(\pi \;\text{and}\;h_{SNP}^{2}\right)$, with $\lambda_{k}=\frac{\sigma_{e}^{2}}{\gamma_{k}\sigma_{g}^{2}}$ and $C_{k}=n+\lambda_{k}$, and Z is a data-dependent random variable following a non-central Chi-square distribution with the non-centrality parameter $NCP=\frac{nv}{\sigma_{e}^{2}}$:

(15) $$Z∼\chi_{1}^{2}\left(\frac{nv}{\sigma_{e}^{2}}\right)$$

Given the threshold of PIP being α for the hypothesis test, the power to detect this causal variant can be calculated as

(16) $$Power_{v}=\text{Pr}(PIP>\alpha |v)=\int_{\alpha }^{1}f(PIP|v)\text{d}\text{PIP}$$

where f(PIP∣v) is Eq (14) above. To compute the power for identifying any causal variant, we integrated out v by

(17) $$Power=\int_{\alpha }^{1}\int_{0}^{\infty }f(PIP|v)f(v)\text{d}v\text{d}PIP$$

where

(18) $$f(v)=f_{\beta }\left(v^{\frac{1}{2}}\right)v^{-\frac{1}{2}}$$

and $f_{\beta }(\cdot )$ is the distribution of β estimated from the SBayesRC model.

Therefore, given a sample size, the expected number of causal variants identified from fine-mapping is

(19) $$E[NCV]=m\left(1-\pi_{1}\right)\times Power$$

The expected proportion of genetic variance explained by the fine-mapped variants is

(20) $$E[PHE]=m\left(1-\pi_{1}\right)\sigma_{g}^{-2}\int_{0}^{\infty }Power_{v}\times vf(v)\text{d}v$$

Since it is computationally challenging to obtain an analytical solution, we opted to estimate these quantities through Monte Carlo simulation (**Supplementary Note**).

### Disease sample size at the liability scale

For diseases or binary traits, we converted the GWAS summary statistics from the linear mixed model to the liability scale prior to running GBMM. Based on the method in Yang et al.^44^, we estimated the sample size at the liability scale that gives an equivalent power to detect a locus affecting a quantitative trait with the same properties,

(21) $$N_{eq}=\frac{i^{2}v(1-v)N_{01}}{{\left(1-K\right)}^{2}}$$

where K is the disease prevalence, v is the sample prevalence, i=h/K with h being the height of standard normal curve at the truncation point t=1−K, and $N_{01}$ is the total number of cases and controls. Given the z-score $\left(Z_{j}\right)$ from the original GWAS summary data for SNP j, the marginal effect and its standard error at the liability scale can be estimated as following ref^55^

(22) $$SE_{j}=\sqrt{\frac{1}{2p_{j}\left(1-p_{j}\right)\left(N_{eq}+z_{j}^{2}\right)}}$$

(23) $$b_{j}=z_{j}\times SE_{j}$$

where $p_{j}$ is the minor allele frequency of the SNP.

The results from GBMM using the converted summary statistics will be directly comparable across traits regardless of the sample prevalence and the type of traits. In our prediction analysis of power, we compared results between diseases and quantitative traits based on the equivalent sample size estimated from Eq (23). Similarly, to estimate the number of cases required, in a case-control study with equivalent number of controls, to achieve a certain power, we rearranged the same equation so that

(24) $$N_{cases}=\frac{1}{2}\frac{{\left(1-K\right)}^{2}N_{eq}}{i^{2}v(1-v)}$$

### Simulations based on imputed genotype data from the UK Biobank

To evaluate the performance of GBMM, we ran simulations using the imputed genotype data from the UK Biobank after quality controls (QC). In this study, we selected 300,000 unrelated individuals and included ~1.2 million HapMap3 SNPs with MAF > 0.01, Hardy-Weinberg equilibrium test *P* > 1×10^−6^, genotyping rate > 0.95, and imputation information score > 0.8 for simulations.

We randomly sampled $m_{c}=10,000$ casual variants from the genome for 100,000 individuals and simulated complex trait phenotypes based on the following model:

(25) y=Xβ+e

where X is the genotype matrix for the causal variants, $\beta_{i}∼N\left(0,h^{2}/m_{c}\right)$ and $e_{j}∼N(0,var(X\beta )/\left(1/h^{2}-1\right))$ with $h^{2}=0.5$ being the proportion of phenotypic variance explained by all the causal variants. To check the robustness of GBMM, we also ran simulations under various settings. For simulations under LD-MAF stratification model, we partitioned all the genome-wide SNPs into four LD and MAF groups (by their median values) and only sampled the 10,000 causal variants in the high LD and high MAF group. For the major gene model simulation, we separated the sampling of effect size for causal variants from two distributions, i.e., 10 random SNPs with effects from $N\left(0,0.2*h^{2}/10\right)$ and the rest SNPs with effects from $N\left(0,0.8*h^{2}/9990\right)$.

We ran a standard GWAS using the genotypes with the simulated phenotypes under different settings. We then used the GWAS summary data to perform GBMM (SBayesRC27 and SBayesC^24^) implemented in GCTB, SuSiE^11^, FINEMAP^12^, SuSiE-inf^13^, FINEMAP-inf^13^ and PolyFun-S^19^ to detect fine-mapped variants and compute corresponding PIPs and effect sizes. We used imputed genotypes from 10,000 random samples from UK Biobank as the LD reference in this study. We repeated the whole process 100 times and then quantified the true discovery rate, mapping precision and replication rate for each method. The mapping precision was computed as the physical distance between the identified SNPs and nearest causal variants.

### Real data analysis

We analysed 598 UK Biobank complex traits GWAS summary data from Neale’s lab (Data Availability) and the schizophrenia^41^ and Crohn’s disease^42^ GWAS summary data. We selected these 598 traits with z-score > 4 and high confidence for heritability estimates using LD score regression. We used the annotations from baseline model BaseLineLD v2.2^38^ and extract the imputed SNPs with MAF > 0.001 and that are in common with the annotations, leading to 13,065,104 imputed SNPs passed the quality control. We used 10,000 random samples from the UK Biobank as the LD reference to run the SBayesRC and other region-specific fine-mapping analysis. We further extracted 48 well-powered traits with relatively large sample size (n > 100, 000), high heritability ($h^{2}>0.1$) and at least a fine-mapped SNP at PIP > 0.9.

For the polygenic score prediction analysis using fine-mapped variants only, we performed quality control for the imputed genotype data provided by the UKB analysis team^36^. We kept SNPs with MAF > 0.01, Hardy-Weinberg Equilibrium test P > 10^−10^, imputation info score > 0.6 within each ancestry samples. We removed samples with mismatched sex information, samples withdrawn from participation and cryptic related samples following ref^27^. We separate the final UKB dataset into 4 ancestries, European (EUR, N= 347,800), East Asian (EAS, N=2,252), South Asian (SAS, N=9,436) and African (AFR, N=7,006). The phenotypes with continuous values were filtered within the range of mean +/− 7SD and then rank-based inverse-normal transformed within each ancestry and sex group. The GWAS were performed using PLINK2 software5 with sex, age and first 10 principal component as covariates. Linear regression was used for continuous traits and logistic regression for binary traits.

## Data Availability

Our SBayesRC-enabled genome-wide fine-mapping results for 600 complex traits are available at link (https://sbayes.pctgplots.cloud.edu.au/data/SBayesRC/share/Finemap/v1.0/). The UK Biobank data are available through formal application to the UK Biobank (http://www.ukbiobank.ac.uk). The GWAS summary data for 598 complex traits in UK Biobank are from http://www.nealelab.is/uk-biobank/. All the other datasets used in this study are available in the public domain.
